# Histologic Evaluation of Soft Tissues around Dental Implant Abutments: A Narrative Review

**DOI:** 10.3390/ma15113811

**Published:** 2022-05-27

**Authors:** Chiara Cinquini, Vincenzo Marchio, Edouard Di Donna, Fortunato Alfonsi, Giacomo Derchi, Marco Nisi, Antonio Barone

**Affiliations:** 1 Department of Surgical, Medical, Molecular and of Critical Area Pathologies, Complex Operative Unit of Stomatology and Oral Surgery, University-Hospital of Pisa, University of Pisa, 56126 Pisa, Italy; chiara.cinquini@gmail.com (C.C.); vincenzomarchio91@gmail.com (V.M.); studiodralfonsi@gmail.com (F.A.); gnolo78@gmail.com (G.D.); marco.nisi@unipi.it (M.N.); 2 Unit of Oral Surgery and Implantology, Division of Oral and Maxillofacial Surgery, Department of Surgery, University Hospitals of Geneva, 1205 Geneva, Switzerland; edo.didonna@hotmail.com

**Keywords:** dental implants, soft tissues, transmucosal surface, abutment

## Abstract

The basis for dental implant success comes not only with the titanium implant osseointegration but also depends on other factors such as the development of a soft tissue barrier, which protects the peri-implant bone from the oral environment. The characteristics of surfaces in contact with peri-implant soft tissues may affect the capacity of peri-implant mucosal cells to create a tight seal around the implant, thus influencing long-term implant success. Many histological studies on animals have been conducted on different materials to better understand their influence on peri-implant soft tissues, with the limitation that results from animal studies cannot be fully translated in humans. The aim of this review paper was to analyze the literature focusing on histological clinical studies in humans which have examined different materials or different surface treatments and their effects on peri-implant soft tissues. The research was conducted according to the following PICO question: “Do different implant/abutment materials affect peri-implant soft tissues adhesion and health?”. Nine articles were analyzed in this review. The results of this review show the influence of different abutment materials on the peri-implant soft tissues, and the need of further research regarding the effect that abutment materials, surface treatments, and surface properties have on soft tissues.

## 1. Introduction

Dental implants are widely used to replace missing teeth with high predictability [1]. The basis for this success lies in the titanium implant osseointegration healing process [2]. The long-term success of dental implants, however, is also related to other factors such as the development of a soft tissue barrier which protects the peri-implant bone from the oral environment, plaque, and oral bacteria [3]. One of the steps of the surgical procedure of implant placement includes the mucosal incision at the implant-receiving site which is then sutured over the fixture or around a transmucosal component, which can be part of the fixture itself or can be represented by a healing abutment or an immediate provisional restoration. It has been demonstrated that, during the healing period, the mucosa forms an attachment to the transmucosal component, de facto separating the bone from the oral environment [4]. Numerous animal studies have been conducted to evaluate the histologic features of peri-implant soft tissues [5,6], showing similarities with the gingival attachment of natural teeth [4]: the peri-implant soft tissues are composed of a connective tissue attachment, which contacts the transmucosal component with collagen fibers parallel to it, and an epithelial attachment [7]. According to the literature, data from animal studies suggest that the peri-implant soft tissues’ height is composed of 1.5–2 mm of epithelium and 1 to 1.5 mm of connective tissue [4,8].

Peri-implant tissues, despite the similarities with the periodontal tissues, possess some unique characteristics, such as the absence of cementum and periodontal ligament, which lead to a direct contact between implant and bone [7].

The peri-implant mucosa structure is based on a keratinized oral epithelium, which continues in the sulcular epithelium [9]. The peri-implant epithelial attachment is histologically similar to the junctional epithelium of natural teeth [10]. The most apical part of the epithelium ends 1–1.5 mm above the peri-implant bone crest, from which it is separated by the connective tissue [10]. 

The peri-implant connective tissue shares some clinical and histological features with natural teeth while also showing some differences, such as the cellular composition and the orientation of the collagen fibers [7]; in fact, this bundle of collagen fibers originates from the periosteum of the alveolar bone and is oriented parallel to the surface of the most coronal part of the fixture [11], while in natural teeth the fibers run perpendicular to the root’s surface, coming into contact with the root cementum. Moreover, the mature peri-implant connective tissue is found to be rich in collagen fibers but poor in cells and blood vessels [12]. 

As previously reported, peri-implant soft tissues are the result of a healing process which takes place immediately after the implant insertion and continues for several weeks [10,13]. 

According to the literature, the establishment and maintenance of peri-implant soft tissues over time are crucial factors for dental implants’ success [12]; the quality of the surfaces in contact with peri-implant soft tissues, whether they are a part of the fixture itself or belong to healing abutments and prosthetic restorations, may affect the capacity of peri-implant mucosal cells to create a tight seal around the implant, thus influencing long-term implant success [14]. 

Several characteristics of transmucosal components may be involved to obtain the mucosal adhesion around the implant: the material characteristics, the surface topography, and implant components and connections [12].

Various materials and their different surface treatments have been extensively studied in vitro to assess their effects on peri-implant soft tissues, focusing on the fibroblast’s activity modulation and on the oral biofilm formation [15,16,17,18,19]. For example, a surface treated with nanoporous titanium dioxide (TiO_2_) may change the reactivity of the implant surface; in fact, results from animal studies showed a decreased inflammatory reaction, a good epithelial attachment, and less marginal bone loss when compared to non-treated implants [20,21]. In addition, some authors reported that chemically modified hydrophilic surfaces have proven to enhance soft tissues’ integration of dental implants [22], and several in vitro studies focused on the capacity of nanostructured hydroxyapatite to enhance epithelial cell attachment [23,24]. Moreover, to improve the soft tissues’ esthetic outcomes, abutments with different colors are used to enhance light reflection, for example zirconia and gold alloy abutments [25]. With regard to the surface roughness, it is still not clear whether a rough surface is preferable to a smooth (machined) surface [26]. According to some authors, a smooth transgingival surface may reduce the bacterial adhesion leading to the reduction of incidence of peri-implantitis [27], while others assert that a rough surface may promote epithelial cell adhesion, thus creating a better soft tissue seal [12]. 

Many histological studies on animals have been conducted on different materials to better understand their influence on peri-implant soft tissues [28], with the limitation that results from animal studies cannot be fully translated into human models and the oral environment [29]. Therefore, the importance of histological clinical studies in humans should be underlined.

The aim of this review paper was to analyze the literature focusing on histological clinical studies in humans which have examined different materials or different surface treatments and their effects on peri-implant soft tissues.

## 2. Materials and Methods

The research was conducted by two groups of reviewers (Group 1: C.C./V.M.; Group 2: A.B./E.D.) based on the following PICO question: “Do different implant/abutment materials affect peri-implant soft tissues adhesion and health?” The reviewers set up the following inclusion/exclusion criteria:

Inclusion criteria:

- human studies
- articles published in English
- randomized clinical trials
- quantitative and/or qualitative histological evaluation of peri-implant soft tissues

Exclusion criteria:

- in vitro studies
- animal studies
- articles published in languages other than English
- non-randomized clinical trials
- book chapters
- review or systematic review articles

Two separate groups of reviewers conducted a double Medline search through PubMed on literature published up to January 2022.

Reviewers of group 1 (C.C./V.M.) conducted the research using the following group of keywords: “transmucosal, surface, implants, soft tissues,” obtaining 70 results. 

Reviewers of group 2 (A.B./E.D.) conducted the research using the group of keywords: “implants, soft tissues, healing abutment, materials, histology,” obtaining 68 results. 

**Figure 1:**
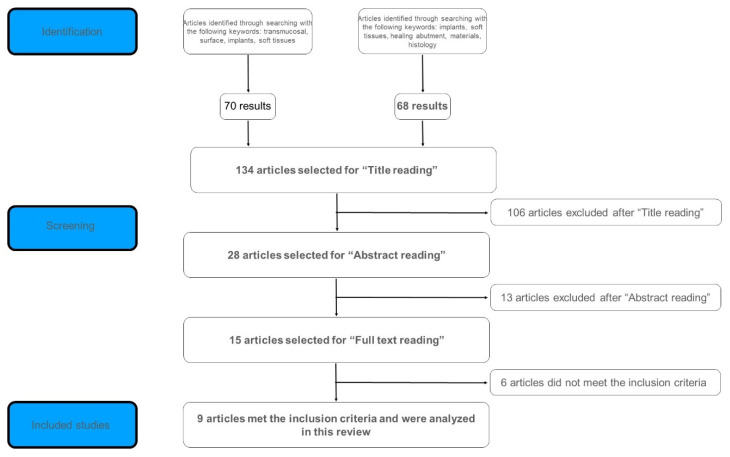
Article selection process.

Once the results were compared and the duplicates were discarded, a total of 134 articles was selected for the title screening. The title screening was performed by both the couples of reviewers, and the results were compared and discussed; of the 134 articles initially included, 28 articles were selected for the abstract screening by both the couples of reviewers, and the results were compared and discussed. After the abstract screening, 13 articles were excluded and 15 were selected for the full text review by all the reviewers, and the results were compared and discussed. Nine articles met the inclusion criteria and were analyzed in this review. The flowchart of the search strategy and article selection process is shown in Figure 1. Excluded studies were either in vivo animal studies, did not have any histological analysis of peri-implant soft tissues, nor evaluated specific tissue management techniques from an histological point of view.

## 3. Results

The nine studies which were selected were published between 2005 and 2021.

The main characteristics and results of the studies included are shown in Table 1.

The first study examined [29] was a randomized clinical trial performed to characterize the peri-implant soft tissues around different one-piece mini-implants. Twelve patients requiring dental implants in the posterior mandible or maxilla were considered eligible. The inclusion criteria were the will to participate to the study and the possibility to place a mini-implant in the distal site of the therapeutic implant, to be removed with a layer of bone and soft tissues 8 weeks after the placement. 

The experimental implants were composed of a conical intraosseous part (length: 6 mm) and a cylindrical transmucosal portion (4 mm), which could be differently treated: machined, acid-etched, or oxidized. Following an eight-week healing period, the implants were harvested with a thin layer of surrounding bone and soft tissues using a soft tissues punch and dedicated trephine burs.

The histologic analyses were performed with the use of a stereomicroscope or a light microscope. 

The height of the peri-implant soft tissue barrier (biologic width) was 4.0 ± 0.8 mm for the oxidized surface (1.6 ± 0.3 mm epithelium and 2.2 ± 0.4 connective tissue), 4.5 ± 0.5 mm for the acid-etched surface (1.4 ± 0.6 epithelium and 2.6 ± 0.6 connective tissue), and 4.1 for the machined surface (2.9 ± 0.7 epithelium and 0.7 ± 0.2 connective tissue).

The implants with oxidized or acid-etched surfaces showed a lower epithelium height but a greater connective tissue height when compared to machined surfaces. No statistical analysis was performed to quantify this difference.

The second study included in the review [30] was conducted to compare the histologic features of two different transmucosal implant surfaces: nanoporous TiO_2_ surface (test group) vs. an unmodified turned surface (control group). Thirty experimental micro-implants were used in this study: according to the gingival thickness, the length of the implants was set at 10 and 13 mm, with a diameter of 2.2 mm. Patients over 18 years having a bone quality permitting the removal of the experimental micro-implants were considered to be eligible for the study.

Fifteen patients received two micro-implants each, one with the test surface and one with the control surface, which were left to heal unsubmerged for 14 weeks and then removed with a trephine bur or with a bone chisel together with a layer of surrounding bone and soft tissues. At the time of removal, no erythema, tenderness, or fluid expulsion was observed in the test group, while signs of inflammation were reported for two patients of the control group. 

The mean percentage of the oral mucosa (intended as epithelium and connective tissue) in contact with the implant transmucosal part was 72% for the test group and 48% for the control group with a statistically significant difference favoring the test group (*p* = 0.027). Considering the epithelium and the connective tissue separately, the percentages were respectively 63/34% for the epithelium and 79/64% for the connective tissue, with no statistically significant difference between the groups. No difference was observed in the median area of the sulcus nor in the height or thickness of the marginal gingiva. Moreover, no difference was observed in the number of inflammatory cells and fibroblasts, and at the ultrastructural examination between the two groups.

In the third study, Degidi and colleagues [31] compared histologically the peri-implant soft tissues around two different transmucosal materials: standard machined titanium or acid-etched titanium healing caps. Eleven patients were enrolled for the study, with a total of twenty-four implants placed in a non-submerged mode. Half of the implants was randomly supplied with standard, machined, prefabricated caps of commercially pure titanium (control), while the other half was randomly provided with acid-etched commercially pure titanium caps (test). After a 6-month healing period, a gingival biopsy was performed around the healing caps of both groups, without unscrewing or removing the caps. The immunohistochemical analysis was performed to evaluate the inflammatory infiltrate, microvessel density, levels of two inflammatory mediators (NOS1, NOS3), vascular endothelial growth factor (VEGF) expression, the proliferative activity (evaluated by the Ki-67 positivity), and the B and T lymphocyte and histiocyte positivity. 

The results showed a larger inflammatory infiltrate in the test group when compared to the control group, with a higher microvessel density and higher expression of VEGF. Additionally, the number of T and B lymphocytes were more important in the test specimens, as well as the Ki-67, NOS1, and NOS3 expression. 

In the fourth study explored, Schwarz and colleagues [32] conducted research to evaluate the performance in terms of the soft tissues’ integration of three different types of healing abutments in humans. Thirty patients aged between 18 and 80 years, needing at least one dental implant in the posterior region of the maxilla or mandible and with good general health were included in this trial. After the implant placement, the implants were randomly allocated to receive one of the experimental healing abutments: Machined titanium (M), hydrophilic acid-etched titanium (modMA1) or titanium–zirconium alloy (modMA2). After 8 weeks, the experimental healing abutments were biopsied obtaining a circular rim consisting of surrounding soft tissues. 

Only 18 healing abutments were analyzed due to various healing complications, including the loss of two implants. The parameters assessed by the histologic analysis were the following: the percentage of soft tissue to abutment contact, biofilm formation, collagen fibers’ orientation, and the presence of inflammatory cells. All the parameters failed to reach statistically significant differences when compared among groups. The authors concluded that the safety and efficacy of modified groups (modMA1 and modMA2) were not inferior for standard machined abutments.

In the fifth study, to evaluate the soft tissues’ immunologic response to standard titanium implants and hydroxyapatite-coated implants, De Wilde and colleagues [33] performed a randomized clinical trial on 13 patients. The inclusion criteria were the need of at least one regular dental implant and a good periodontal health. The one-piece mini-implants (diameter 1.5 mm and length 8 mm) were inserted according to a randomization scheme; one week after surgery the crevicular fluid was sampled using paper points and, eight weeks after the insertion, the mini-implants were removed with a layer of surrounding bone and soft tissues and were replaced by regular implants. The mRNA was extracted by the peri-implant crevicular fluid and analyzed through the selection of the following markers of inflammatory response: growth factor beta-2 (TGF-ß2), collagenase-2 (MMP-8), chemokine ligand-3 (CCL-3), interleukin-8 (IL-8), IL-1 ß, and IL-6. The specimens’ histological sections were analyzed with an image analysis software for histomorphometric evaluation. The histologic analysis was performed only on 10 readable paired samples: the authors observed that the morphology of the soft tissues implant interface varied considerably among patients, with different connective tissue/epithelium ratios. Inflammatory cells were present in both groups (test and control) and no statistically significant difference was observed for the histomorphometric analysis. Moreover, according to the RNA analysis, no significant differences was found in the immunological response to the two different types of implants.

In the sixth study, Garcia and colleagues (Garcia et al., 2016) [34] designed a randomized clinical trial to investigate the influence of plasma cleaning on the interaction between soft tissues and abutments. Thirty healthy patients who needed an implant rehabilitation for a single tooth were included in the study, and each received 30 submerged implants 4.8 mm in diameter. At the second surgery, fixtures were uncovered, and specially designed abutments were placed. At this time, patients were randomly allocated to one of two groups: the standard abutment group (15 patients) and the plasma-of-argon-cleaned abutment group (15 patients). After 2 weeks, a special punch was used to biopsy the soft tissues around the abutments, which were removed as one piece (punch-abutment soft tissues’ sample). The samples were then processed in order to be observed under the polarized light microscope. The authors observed that the area occupied by cells was higher in the plasma-cleaned-abutments group than in the no-treatment group in a statistically significant manner (*p* < 0.05). No bacterial contamination was observed in samples from the test group, while it was found in 40% of the samples from the control group. With regard to collagen fiber density, it resulted in higher levels in the test group than in the control group, with a predominance of oblique fibers.

With respect to the seventh study, published in 2017, Sampatanukul and colleagues assessed, in a randomized clinical trial, the histological features and inflammatory responses of soft tissues around three different abutment materials [35]. Healthy, non-smoker patients requiring an implant rehabilitation in the posterior maxilla or mandible were considered eligible for the study. Ten patients underwent implant insertion (15 implants) and were randomized to receive one of three experimental abutments: patients allocated to group one received titanium abutments, patients from group two received zirconia abutments, while patients allocated to group three received gold alloy abutments. Eight weeks after surgery, the biopsy was performed, and the abutments were removed with a layer of soft tissues.

Before the biopsy, the soft tissues’ attachment was evaluated by the gingival index (GI) score [36] with values ranging from zero (pink gingiva) to three (markedly red or reddish, enlarged, ulcerated), by the surgical score (ranging from score one, representing a firm tissue with no detachment, to score three, characterized by a full detachment and a loose tissue), by the histological attachment percentage, by the inflammatory extent grade with values ranging from one to three (a semi-quantitative score attributed according to the amount and location of inflammatory cells in each specimen), and by the inflammatory cellularity grade, which depends on the inflammatory cell density (sparse, moderate, or dense). A total of 15 implants was analyzed. No difference among the groups was detected for GI score and surgical score. With regard to the percentage of soft tissues’ attachment, the gold alloy showed a statistically significant lower percentage (54.66%) when compared to titanium (80.80%) and zirconia (80.12%) abutments. The comparison of titanium and zirconia abutments showed non-statistically significant differences in terms of clinical signs of inflammation, soft tissue attachment, and inflammatory response, whilst the inflammatory response degree was higher in the gold alloy abutment group compared with the titanium group.

In the eighth study, Mangano and colleagues (Mangano et al., 2018) designed a randomized controlled clinical trial with the aim of investigating the effects on peri-implant soft tissues, at an immunohistochemical level, of machined and Direct Metal Laser Sintered (DMLS) healing abutments in different configurations (full machined, full DMLS, and Half DMLS/Half machined). The objective of the study was to evaluate the degree of cell adhesion (through integrin expression) and the quantity/quality of inflammatory infiltrate (based on the expression of CD3 T and CD20 B Lymphocytes and CD68 macrophages). Fifty healthy patients needing an implant rehabilitation of a single tooth were included, and received one submerged implant fixture each. At the second surgery, they were randomly allocated to one of four groups: DMLS healing abutment (11 patients), Machined Upper Half/FMLS Lower Half healing abutment (10 patients), DMLS Upper Half/Machined Lower Half (19 patients), Machined healing abutment (10 patients). After 30 days, gingival biopsies 1.5 mm wider than the healing screw were performed: the average thickness and height of the samples were 2.1 and 3 mm respectively. The samples were then processed and observed using a light microscope. Immunohistochemical analysis demonstrated the presence of adhesion molecules (integrins) between healing abutments and peri-implant tissues: the DMLS and half-DMLS abutments showed a greater presence of integrins than the Machined abutments. All samples were positive for inflammatory infiltrate, with a statistically significant (*p* < 0.05) infiltrate found in DMLS healing abutments when compared to Machined abutments.

In the ninth study, Canullo and colleagues (Canullo et al., 2021) published the results of a randomized controlled clinical trial with the purpose of evaluating the effect of different abutment surfaces on soft tissue morphogenesis and integration. Thirty-six healthy patients needing an implant rehabilitation were included in this study: each patient received one submerged implant fixture and, at the second surgery, patients were randomly assigned in four groups, receiving a specially designed abutment with one of four different surfaces: Smooth-surface-machined (MAC, eight patients and one dropout), Ultrathin Threaded Microsurface (UTM, nine patients), Smooth-surface-machined and plasma-of-argon-activated (PLASMA-MAC, nine patients) and Ultrathin Threaded Microsurface and plasma-of-argon activated (PLASMA-UTM, 9 patients). After 60 days, 1.3 mm diameter gingival biopsies were obtained with a specially designed 5 mm-wide tissue punch which allowed the removal of abutment, sample, and the tissue punch as one piece. The samples were then processed and observed through a polarized light microscope. 

Tissues in contact with MAC surfaces showed an improved morphogenesis and those in contact with surfaces which underwent plasma of argon treatment showed good quality connective tissue particularly in thin tissues, with an increased ratio between the thickness of connective compartment and epithelium. 

Approximately two-thirds of MAC and UTM cases showed a moderate or severe grade of inflammation when compared to PLASMA-MAC and PLASMA-UTM surfaces, which exhibited a moderate/severe inflammation only in one-third of the cases. Erythematous/exudative process (an indicator of inflammation) was absent in the PLASMA-MAC group and was observed in all the other groups. The significant performance of plasma groups in terms of soft tissues’ behavior is visible only in thin preoperative biotypes (less than 2 mm), while it has no influence on thick preoperative biotypes (more than 2 mm).

**Table 1:** Main characteristics of the included studies.

Reference	N° of Patients	N° of Implants/Abutments	Experimental Groups	Harvesting Procedure	Histologic Analysis	Results
**Glauser et al. (2005)** [29]	5	12 titanium mini-implants	Group 1 (4) = oxidized surface Group 2 (4) = acid-etched Group 3 (4) = machined surface	Implants were harvested with a layer of surrounding hard and soft tissues Healing period: 8 weeks	Stereomicroscope/light microscope	Oxidized and acid-etched surface showed a lower epithelium height but a greater connective tissue height when compared to machined surface
**Wennerberg et al. (2001)** [30]	15	30 titanium micro-implants	Control Group (15) = unmodified turned surface Test Group (15) = nanoporous TiO_2_ surface	Implants were harvested with a layer of surrounding hard and soft tissues. Healing period: 14 weeks	Light microscope/Transmission Electron Microscopy	The mean percentage of oral mucosa in contact with the implant transmucosal part was 72 and 48%, respectively for the test and the control group, with a statistically significant difference (*p* = 0.027)
**Degidi et al. (2012)** [31]	11	24 implants/24 healing abutments	Control Group (12) = Standard machined, prefabricated titanium caps Test Group (12) = Acid-etched titanium caps	Gingival biopsies 5.5 mm diameter around healing cap surface Healing period: 24 weeks	Light microscope	Tissues around acid-etched titanium caps (test) showed a higher rate of restorative processes which is correlated with a higher inflammation processes observed in these tissues.
**Schwarz et al. (2013)** [32]	30	18 implants/18 healing abutments	M (5) = machined modMA1 (6) = hydrophilic acid etched Ti modMA2 (7) = Ti-zirconium alloy	Healing abutments were harvested with a layer of surrounding soft tissues. Healing period: 8 weeks	Light microscope	No statistically significant differences in terms of percentage of soft tissue to abutment contact, biofilm formation, collagen fibers orientation, and the presence of inflammatory cells.
**De Wilde et al. (2015)** [33]	13	25 mini-implants	Control group (13) = Commercially Pure Ti Test group (12) = Nano-hydroxyapatite coated	Implants were harvested with a layer of surrounding hard and soft tissues. Healing period: 8 weeks	Light microscope/image analysis software	No statistically significant differences in the presence of inflammatory cells nor in the expression of inflammatory mediators.
**Garcia et al. (2016)** [34]	30	30 submerged titanium implants	Control group (15) = standard abutments Test group (15) = plasma-of-argon-cleaned abutments	Special punch for biopsy of soft tissues around abutment. Then abutment is disconnected and replaced with standard healing abutment Healing period: 2 weeks	Polarized light microscope	Test group: Higher area occupied by cells, no bacterial contamination, higher collagen fiber density Group control: Bacterial contamination in 40%
**Sampatanukul et al. (2017)** [35]	10	15 implants/15 healing abutments	Group 1 (5) = titanium abutment Group 3 (5) = zirconia abutment Group 3 (5) = gold alloy abutment	Healing abutments were harvested with a layer of surrounding soft tissues Healing period: 8 weeks	Light microscope	The inflammatory response degree tended to be higher with the gold alloy abutment compared to the titanium abutment. Titanium and Zirconia abutments promoted better attachment percentages compared to gold alloy abutments
**Mangano et al. (2018)** [14]	50	50 implants/abutments	T1 GROUP (11): Healing Abutment with Direct Metal Laser-Sintered Surface T2 GROUP (10): Healing abutment with smooth upper half and DMLS lower half T3 GROUP (19): Healing abutment with DMLS upper half and smooth lower half T4 GROUP (10): Healing abutment completely smooth	Gingival biopsies 1.5 mm wider than the healing screw thickness of average 2.1 mm (5.5 mm-3.8 mm) height of 3 mm Healing period: 4 weeks	Light microscope	Immunohistochemical analysis demonstrated the presence of adhesion molecules (integrins) between the HA and the peri-implant tissues. In HA with DMLS surface the presence of integrins is significantly greater than those found on the Machined surface. All samples were positive for inflammatory infiltrate (CD3 T lymphocytes, CD20 B Lymphocytes, CD68 Macrophages): statistically significant lower infiltrate found in HA with DMLS surface compared to smooth surface.
**Canullo et al. (2021)** [28]	36	36 implants	MAC (smooth-surface-machined) 9-1 drop out, 8 SAMPLES UTM (Ultrathin threaded microsurface) 9 Plasma-MAC (MAC plasma-of-argon-activated) 9 Plasma-UTM (UTM plasma-of-argon-activated) 9	Gingival biopsies with a 5 mm wide punch. Peri-implant collar of tissue of about 1.3 mm removed with the abutment. Healing period: 8 weeks	Polarized light microscope	MAC surfaces showed to have the ability to improve soft tissues morphogenesis. Moreover, plasma of argon treatment showed a positive effect especially on the connective tissue portion of thin tissues, increasing the ratio between the thickness of connective compartment and epithelium. Approximately 2/3 of MAC and UTM cases showed a moderate or severe grade of inflammation, compared to PLASMA-MAC and PLASMA-UTM surfaces, which exhibited a moderate/severe inflammation only in 1/3 of the cases. Erythematous/exudative process (indicator of inflammation) was absent in PLASMA-MAC group and was observed in all the other groups. The significant performance of plasma groups in terms of soft tissues behavior is visible in case of thin preoperative biotype (less than 2 mm), while it is not in case of thick preoperative biotype (more than 2 mm).

## 4. Discussion

Titanium is the most favorable material to produce dental implants, because of its biocompatibility and good clinical performances [37], and because it facilitates osseointegration [38]. However, in addition to osseointegration, the quality of soft tissues is also involved in the success and stability of dental implants over time, leading to the concept of soft tissues’ integration [39]. As previously reported, the chemical and topographic features of the external surface of transmucosal implant components are important in the biological response of peri-implant soft tissues [40].

Over the years, various materials have been used to fabricate implant transmucosal part or abutments, such as titanium, gold alloy, or zirconium [41].

According to the results of our review, many modifications to the transmucosal part of implants or abutments may play a role in improving the formation of a firm soft tissue barrier.

The findings of Glauser and colleagues [29] showed that an acid-etched surface or a microporous surface oxidized with TiO_2_ led to the achievement of a longer connective tissue seal when compared to a standard machined transmucosal part, and a lower junctional epithelium height; however, no statistical analysis was performed to quantify this observed difference, thus the results of this study have little scientific value. The authors concluded that the peri-implant soft tissues formed were similar to those observed in animal models. Moreover, it remains unclear as to whether these characteristics could bring an improvement of the soft tissue seal among the three types of abutments used in this trial. These observations could be confirmed by other authors [30] who conducted a randomized clinical trial to assess the soft tissue response and the percentage of the oral mucosa (epithelium and connective tissue) in contact with the abutment for TiO_2_ microporous surfaces when compared to an unmodified titanium surface. In fact, according to the authors, the TiO_2_ microporous surface showed a significantly higher percentage of oral mucosa contact when compared to an untreated surface (72 vs. 48%, *p* = 0.027), whilst no difference was found in the mean number of inflammatory cells or the numbers of fibroblasts. These findings could suggest a possible role of TiO_2_ microporous surfaces in improving soft tissues’ attachment to implants, with no difference in terms of inflammation response when compared to unmodified titanium.

The study conducted by Degidi and colleagues [31] showed a higher degree of inflammation in peri-implant tissues around acid-etched titanium caps when compared to standard titanium machined caps. 

The surface properties of transmucosal abutment are decisive for bacterial adhesion. These findings may be related to a higher amount of bacteria colonizing acid-etched abutments, which may be critical for the long-term survival rate of dental implants.

Schwarz and colleagues [32] studied the effects f soft tissues of three different abutment materials: machined titanium, hydrophilic-acid-etched titanium, and titanium–zirconium alloy. These effects were evaluated in terms of the percentage of soft tissue contact, biofilm formation, collagen fibers’ orientation, and the presence of inflammatory cells. No differences were observed among the three different abutments for all the parameters registered. The authors concluded that safety and efficacy were similar in all the three groups, since all the adverse events observed in the healing period were equally distributed among the groups. Moreover, even if the element is not statistically significant, the authors observed an improvement in soft tissues quality around the modified abutments (acid-etched and titanium–zirconium) and a major percentage of perpendicular collagen fibers at the soft tissues’ abutment interface. However, the clinical relevance of these findings still has to be clarified.

De Wilde and colleagues (De Wilde et al., 2015) conducted a study to evaluate the soft tissues’ immunologic response to implants coated with or without nano-sized hydroxyapatite coatings. In this study, no statistically significant difference was observed for the histomorphometric analysis. Moreover, based on RNA analysis, no significant differences were found in the immunological response to the two different types of implants. According to this study, the nanocoating that is normally used to improve osseointegration can also be applied as an abutment coating.

The study published by Garcia and colleagues (Garcia et al., 2016) indicates that the plasma-cleaning procedure on abutments had positive effects on soft tissues’ interaction with a greater cell-occupied surface area and led to significantly less bacterial contamination than in untreated abutments. Additionally, the collagen fibers were predominantly arranged in an oblique position and with a higher density. 

Another study [34] conducted on three different abutment materials (titanium, zirconia, and gold alloy) showed comparable percentages of soft tissue attachment in titanium and zirconia abutments, while the gold alloy showed a significantly lower percentage of soft tissue attachment and a higher degree of inflammation; however, no statistical difference in the Gingival Index score was observed among the groups.

The authors concluded that titanium and zirconia abutments promoted the achievement of a better soft tissue condition when compared to gold alloy abutments.

Mangano et al. [14] conducted a trial in humans to evaluate soft tissue adhesion and inflammatory response on Direct Metal Laser Sintered (DMLS) Healing Abutments in different configurations: full DMLS, half DMLS and half machined, and full machined.

The arrangement of peri-implant collagen fibers plays a role in the stability and strength of the connective tissue attachment to the implant neck and overlying prosthesis. A very recent randomized controlled clinical trial from Canullo and colleagues (Canullo et al., 2021) indicated that smooth surface machining (MAC) improved soft tissues’ morphogenesis around implants, and that the plasma of argon treatment had a significant effect especially on the connective tissue portion of thin tissues.

The results obtained by this narrative review of the literature show that further research regarding the effect that abutment materials and surface treatments have on soft tissues is needed: each study included in the assessment provided unique ideas and points of view on the evaluation of peri-implant soft tissues, but offered only a few common threads. 

First of all, histological human studies on peri-implant soft tissues need to be performed using specially designed soft tissue punches which allow for the retrieval of the soft tissue–abutment complex as a singular, encapsulated unit. This would provide a precise histologic analysis of the relation between soft tissues and abutment. 

Another important factor are the outcomes that need to be considered to evaluate the peri-implant soft tissues, such as: 

epithelium height

- connective tissue height
- percentage of the oral mucosa in contact with the implant transmucosal portion
- biofilm formation and bacterial contamination
- collagen fibers’ orientation and density
- presence of inflammatory cells
- expression of inflammatory mediators and inflammatory infiltrate (CD3 T lymphocytes, CD20 B lymphocytes, CD68 macrophages)
- evaluation of the attachment to the abutment’s surface through the measurement of integrins expression.

The results of this review show the influence of different abutment materials on the peri-implant soft tissues. After reviewing the literature, only nine studies on humans were reported (Table 1) and this draws attention to the lack of histological clinical studies in humans. According to these studies, titanium and zirconia abutments promoted a better tissue attachment percentage when compared to gold alloy abutments. Chemically treated surfaces of titanium abutments such as oxidated, acid-etched, nanoporous titanium dioxide, or plasma of argon cleaning have shown several beneficial effects when compared to other types of surfaces. These positive effects have been identified in the patients with a greater connective tissue height and thickness: moreover, the density of collagen fibers and predominance of oblique fibers have been observed in these patients. 

However, results found by Degidi and colleagues [31] suggest caution in roughening the abutment surface. In fact, if the rougher part of the abutment becomes exposed to the oral cavity, the risk of a higher degree of inflammation in the peri-implant tissues might increase.

## 5. Conclusions

The conclusions that can be drawn from this narrative review of the literature are twofold.

First, a model for the histologic study of peri-implant soft tissues in humans and how abutment materials influence them can be determined. The analysis of the listed outcomes through the histomorphometric analysis of peri-implant soft tissues samples might help determine, as previously stated, which combination of abutment material characteristics is ideal for obtaining the soft tissue seal. An interesting factor to investigate, which was not considered in the studies included in the review, is the abutment shape and its influence on peri-implant soft tissues.

Second, from a clinical standpoint and based on the observations of the studies collected in this review, special care must be taken in choosing the abutment material and surface treatment: at the very least, plasma of argon cleaning should be routinely chosen for its beneficial effects on the surrounding tissues. In addition to choosing the right material and surface treatment, tissue morphology needs to also be considered: greater tissue height and thickness seem to be favorable towards obtaining healthy and stable peri-implant soft tissues, which may suggest performing connective tissue grafting in patients with insufficient soft-tissues thickness.

These findings are very interesting for daily practice and for future research; the limited number of studies and number of patients included, however, requires further studies to substantiate these findings and to appreciate their magnitude. To investigate the fine line between “too much” and “just enough” abutment surface roughness, the influence of abutment shape on soft tissues, and an increase in the number of histologic studies on human subjects are fundamental to determine which is the gold standard for obtaining ideal peri-implant soft tissues’ morphology and sealing ability.

## Data Availability

Not applicable.
